# Tailored Nanoparticles With the Potential to Reduce Ruminant Methane Emissions

**DOI:** 10.3389/fmicb.2022.816695

**Published:** 2022-03-11

**Authors:** Eric Altermann, Kerri Reilly, Wayne Young, Ron S. Ronimus, Stefan Muetzel

**Affiliations:** ^1^AgResearch Ltd., Palmerston North, New Zealand; ^2^Riddet Institute, Massey University, Palmerston North, New Zealand; ^3^School of Veterinary Science, Massey University, Palmerston North, New Zealand

**Keywords:** bionanoparticles, methane mitigation, biotechnology, sustainable agriculture, climate change, methanogens, lytic enzymes, rumen

## Abstract

Agricultural methane produced by archaea in the forestomach of ruminants is a key contributor to rising levels of greenhouse gases leading to climate change. Functionalized biological polyhydroxybutyrate (PHB) nanoparticles offer a new concept for the reduction of enteric methane emissions by inhibiting rumen methanogens. Nanoparticles were functionalized *in vivo* with an archaeal virus lytic enzyme, PeiR, active against a range of rumen *Methanobrevibacter* species. The impact of functionalized nanoparticles against rumen methanogens was demonstrated in pure cultures, in rumen batch and continuous flow rumen models yielding methane reduction of up to 15% over 11 days in the most complex system. We further present evidence of biological nanoparticle fermentation in a rumen environment. Elevated levels of short-chain fatty acids essential to ruminant nutrition were recorded, giving rise to a promising new strategy combining methane mitigation with a possible increase in animal productivity.

## Introduction

Climate change presents an increasingly serious threat to all ecosystems (Overpeck and Udall, 2020; Soroye et al., 2020; Tabari, 2020; Witze, 2020). Although the contribution of anthropogenic emissions, and in particular ruminant methane emissions has been recognized for decades (Johnson, 1974; Sawyer et al., 1974; Burch, 2020; Ku-Vera et al., 2020; Ribeiro et al., 2020; Wolski et al., 2020), pressure on agricultural systems, the need to balance land use and food prices, coupled to an increasing demand to feed a growing population limit the ability to reduce animal numbers (Bustamante et al., 2014; Stevanovic et al., 2017; White and Hall, 2017). Enteric methane emissions predominantly stem from the microbial fermentation of feed in the forestomach of ruminants where a specialized group of archaea, commonly known as methanogens, converts free hydrogen through the reduction of carbon dioxide. The rate of methane production and methanogen growth rates are dependent on a range of factors such as free and dissolved hydrogen, pH, and ruminal passage rates (Janssen, 2010). Methane is the second largest driver of global radiative forcing (Stocker et al., 2013) and there is a growing and urgent need to reduce enteric methane emissions without negatively impacting on ruminant productivity, food quality or food safety.

A range of strategies is currently being pursued to mitigate enteric methane emissions aiming to reduce the number or metabolic activity of rumen methanogen and include such diverse concepts as farm system changes (Chiavegato et al., 2015), animal genetics and breeding (Gonzalez-Recio et al., 2020), forage optimization (Warner et al., 2017), diet interventions (Borsting et al., 2020), methanogen inhibitors (Hristov et al., 2015), vaccines (Subharat et al., 2016), and natural additives (Belanche et al., 2020).

While some of these approaches show promise, they also come with drawbacks including seasonality of feed, cost of manufacture and distribution, scalability to farm levels, and the prolonged time of development and lack of successors that may be outpaced by rapid microbial adaptation.

Phage therapy holds such a premise in principle (Reardon, 2014) and is a well-recognized and increasingly important medical strategy in light of widespread antibiotic resistance. However, the complexity of phage, their limited host range and challenges in biotechnological upscaling (Skurnik et al., 2007) currently limit a more widespread use. In contrast, phage lysins, enzymes capable of hydrolyzing bacterial peptidoglycan, provide a range of distinct advantages over intact phage and are under development as a priority antibiotic alternative (Fischetti, 2018). Emerging evidence demonstrates that lysins of archaeal viruses are similarly capable of hydrolyzing pseudomurein, a cell wall type common to the main methanogen clades in the rumen (Schofield et al., 2015).

Free enzymes are often not as stable as desired and a highly active ecosystems such as the rumen with a high protein and substrate turnover, would potentially limit the opportunity for free lytic enzyme activity before degradation. Enzymes immobilized on a solid substrate are known to be much more resilient toward physical and biological stresses (Ansari and Husain, 2012) and can be effectively used in continuous flow bioreactors (Chandra et al., 2020). However, the orientation of enzymes, i.e., their physical alignment in relation to the surface of the immobilization matrix within these systems, is likely to be random and may reduce the effectiveness of these systems by sterically preventing enzyme-substrate interactions.

Functionalized biological nanoparticles comprised of polyhydroxyalkanoates (PHA) hold the promise of combining the advantages of enzyme immobilization while enhancing the technology with directional enzyme display on the bead surface and a one-step biosynthesis (Jahns and Rehm, 2009). The enzyme catalyzing the polymerization of hydroxybutyrate to polyhydroxybutyrate (PHB), PHB synthase PhaC (Yuan et al., 2001), remains covalently bound to its own polymer while accepting both N- and C-terminal protein fusions without activity loss. This property has enabled the directional display of enzymes on the nanoparticle surface (Rehm, 2010).

Combining the well-known systems of phage therapy with emerging and scalable biotechnology holds the promise of a novel and flexible concept to mitigate enteric methane emissions. For example, two archaeal lytic enzymes, PeiP and PeiW have been extensively characterized (Visweswaran et al., 2010; Schofield et al., 2015) and, as members of the C71 peptidase family, hydrolyze the pseudomurein sacculus of Methanobacteriales and Methanopyrales as monomers. In previous research, we have successfully demonstrated that by displaying the lytic enzyme of a *Methanobrevibacterium ruminantium* M1 virus (Leahy et al., 2010) on the surface of PHB nanoparticles, effective inhibition of cell growth and methane production was possible in pure cultures of a range of *Methanobrevibacter* strains (Altermann et al., 2018). In contrast to PeiP and PeiW, PeiR is biochemically less characterized and belongs to a different enzyme family (C39 peptidase).

We developed the hypothesis that tailored nanoparticles are capable of inhibiting methane in complex biological systems such as the rumen. To test the effectiveness of lytic-enzyme-nanoparticles in a rumen simulation, we have developed dual-fusion nanoparticles displaying two lysins per PhaC enzyme and evaluated their ability to reduce methane emissions in rumen batch and continuous flow fermentations.

## Materials and Methods

### Microbial Strains and Culture Conditions

*Methanobrevibacter ruminantium* M1 (DSM 1093) and *Methanobrevibacter* sp. D5 were grown anaerobically from frozen stocks in Hungate tubes in 10 ml of RM02 medium supplemented with 5% rumen fluid, yeast extract (0.1%), calcium chloride (4 mM), and magnesium chloride (4 mM) as described elsewhere (Kenters et al., 2011). Vitamin 10 solution (0.1 ml per 10 ml culture tube) (Joblin et al., 1990), methanol (20 mM), sodium formate (60 mM), sodium acetate (20 mM) and coenzyme M (CoM) (10 μM) were filter sterilized into the medium after autoclaving (Leahy et al., 2010; Wedlock et al., 2010). For growth assays, growing methanogen cultures were anaerobically subcultured into fresh supplemented RM02 medium and incubated at 39°C without agitation in Hungate tubes (Wedlock et al., 2010). The headspace was pumped with hydrogen to 1.4 bar to facilitate methanogen growth.

### Plasmid Construction for PhaC and PhaC-PeiR BNP Production

Production of the pKRpeiR plasmid has been described previously (Altermann et al., 2018). Briefly, the gene encoding PeiR was codon optimized and synthesized by GeneArt^1^ and subsequently amplified with primers containing XhoI and BamHI restriction sites (PeiR-Fwd 5′-CTCGAGATGGTTCGTTTTAGCCGTGATATGC-3′, PeiR-Rev 5′-GGATCCTTATGCCGGACACACAACATAATAATTCTGG-3′). The resulting PCR amplicon was gel purified, digested and ligated into the pET14 plasmid (Novagen), digested with XhoI and BamHI. Resulting clones were isolated and plasmids sequenced for verification. Plasmid pKRpeiR was used for the single gene fusion. The dual fusion plasmid was created by cloning an additional copy of the *peiR* gene upstream of the PhaC–PeiR fusion harbored in pKRpeiR. The peiR gene was amplified from the codon optimized synthetic construct using primers introducing XbaI and NdeI restriction sites (PeiR-N-Fwd 5′-TCTAGACGAAGAAGA TACTAGTCACCATGGTTAGATTCAGCAGAGAC-3′, PeiR-N-Rev 5′-TCATGCAGGACAGACAACATAGTAGCATATG-3′). PCR amplification conditions and subcloning were carried out as previously described (Altermann et al., 2018). The resulting single and dual-fusion plasmids pKRpeiR and pKR-dual-peiR were then transformed into *E. coli* BL21 (DE3) competent cells, which harbored the helper plasmid pMCS69 (Amara and Rehm, 2003). Non-functionalized PhaC control beads were produced with the plasmid pETC (Peters and Rehm, 2008), which encodes the wild-type PhaC enzyme. pETC was transformed into *E. coli* BL21 (DE3) competent cells containing the plasmid pMCS69.

### Production and Purification of Non-functionalized PhaC, Single-Fusion and Dual-Fusion PeiR Nanoparticles

For small-scale pure culture experiments, *E. coli* BL21 (DE3) containing the helper plasmid pMCS69 and either one of the pKRpeiR-C, pKR-dual-peiR, or pETC plasmids were grown aerobically in LB broth supplemented with ampicillin (50 μg ml^–1^), chloramphenicol (64 μg ml^–1^) and 1% (w/v) glucose at 37°C. When the OD600 reached 0.5, nanoparticle production was induced by addition of 1 mM IPTG followed by agitation at 19°C for 48 h. Nanoparticle-producing cells were pelleted by centrifugation at 6,000 × g for 5 min at 4°C, resuspended in 50 mM phosphate buffer (pH 7.5) and lysed via a Microfluidizer (Microfluidics, MA) for 5 min at 4°C through a Z diamond interaction chamber at 1,447 bar. The resulting nanoparticle slurries were purified by ultracentrifugation over a glycerol gradient as previously described (Brandl et al., 1988). Briefly, lysed cells were added to a 2-step glycerol gradient of 44% (v/v) glycerol in 50 mM phosphate buffer pH 7.5 layered on 88% (v/v) glycerol in 50 mM phosphate buffer and centrifuged at 35,000 rpm for 2 h at 4°C in a Sorvall TH641 swing-out rotor (Thermo Fisher Scientific, Auckland, New Zealand) in a Sorvall RC100 ultracentrifuge. Nanoparticles were recovered from the gradient interface by pipetting and were resuspended in 20 mM 3-(N-morpholino)propanesulfonic acid (MOPS, pH 7.0) containing 1 mM DTT, 0.3 M NaCl and 20% glycerol (v/v) and stored at −80°C. Nanoparticles were stained with Nile Red as described previously (Peters and Rehm, 2005) and visualized by fluorescence microscopy using a Leica DM2500 microscope (Leica, Wetzlar, Germany) with UV filter (excitation BP 355–425 nm).

Large-scale preparations of nanoparticles were prepared by growing bulk volumes of respective bacterial cultures carrying both helper and production plasmids grown and spun down (6,000 × g, 30 min, 4°C) and the resulting pellets frozen at −20°C. When enough cell pellet had accumulated, pellets were thawed, resuspended in 50 mM phosphate buffer pH 7.5 and passed through the microfluidizer interaction chamber 10 times at 1,447 bar before pelleting the BNP’s (8,000 × g, 30 min, 4°C) and other insoluble material and resuspending in in 20 mM MOPS pH 7.0 containing 1 mM DTT, 0.3 M NaCl and 20% glycerol (v/v) at a concentration of 500 mg ml^–1^ (wet weight). Beads were dispensed in 5 g lots into 10 ml syringes and stored at −80°C until added to the continuous flow fermenter.

### Methanogen Growth Studies and Methane Measurements

Growing methanogen cultures were subcultured into fresh RM02 medium (Leahy et al., 2010) with substrates in Hungate tubes using a 1% (v/v) inoculum. The culture headspace (CO_2_) was flushed with a H_2_/CO_2_ (80:20 v/v) before pressurizing to 1.4 bar. PhaC (negative control), PhaC-PeiR or Dual-PeiR (treatment) nanoparticles were added at the required dosage and cultures incubated as described above. Cell growth was monitored every 24 h for up to 5 days by measuring OD600 using a Spectronic 200 (Thermo Fisher Scientific, Auckland, New Zealand), directly inserting the tubes into the spectrophotometer and corrected as previously described (Altermann et al., 2018).

The amount of methane present in the headspace was measured at 24 h intervals by gas chromatography using a Varian Aerograph 660 instrument (Walnut Creek, CA, United States) and calculated according to Equation 1. Immediately after gas samples were taken, the headspace was flushed with hydrogen to reset the methane partial pressures and to provide sufficient levels of hydrogen for methanogen growth. All experiments were performed in biological triplicates.


$$C\,H_{4}\,\left[m\,l\right]=\frac{\frac{P\,e\,a\,k\,H\,e\,i\,g\,h\,t_{\left(s\,a\,m\,p\,l\,e\right)}}{V\,o\,l\,u\,m\,e\,L\,o\,a\,d\,e\,d_{\left(s\,a\,m\,p\,l\,e\right)}}*\frac{A\,t\,t\,e\,n\,u\,a\,t\,i\,o\,n_{\left(s\,a\,m\,p\,l\,e\right)}}{A\,t\,t\,e\,n\,u\,a\,t\,i\,o\,n_{\left(s\,t\,a\,n\,d\,a\,r\,d\right)}}*V\,e\,s\,s\,e\,l\,G\,a\,s\,V\,o\,l\,u\,m\,e}{\frac{A\,v\,e\,r\,a\,g\,e\,P\,e\,a\,k\,H\,e\,i\,g\,h\,t_{\left(s\,t\,a\,n\,d\,a\,r\,d\right)}}{V\,o\,l\,u\,m\,e\,L\,o\,a\,d\,e\,d_{\left(s\,t\,a\,n\,d\,a\,r\,d\right)}}*\frac{100}{C\,o\,n\,c\,e\,n\,t\,r\,a\,t\,i\,o\,n_{\left(s\,t\,a\,n\,d\,a\,r\,d\right)}}}$$

**Equation 1:** Calculation of the amount of methane (CH_4_) in a gas sample. Samples were taken from Hungate tubes using gas tight syringes where the pressure in the syringe was equal to the pressure in the Hungate tube. The gas pressure in the syringes was not released before injection into the detector. PeakHeight: height of the corresponding CH_4_ peak in the gas chromatography analysis; VolumeLoaded: Gas volume loaded into the detector (ml); AveragePeakHeight: Average Peak Height of a known standard; VesselGasVolume: Vessel Gas Volume (ml); Concentration: concentration of standard (%).

### Rumen Fluid Collection

The collection and processing of rumen fluid was carried out as described previously (PONE-D-21-05378, see attached file, under review). Briefly, Rumen fluid was collected from two fistulated cows and filtered through a layer of cheese cloth (Stockinette; Cirtex Industries Ltd., 229 Thames, New Zealand). Immediately after filtering, rumen fluid was added to each of the vessels through the feeder inlet. The maintenance of the cannulated cows for rumen fluid collection was approved by the Grasslands Animal Ethics Committee, Palmerston North, New Zealand under the AE number 15154.

### *In vitro* Batch Culture Incubations

The batch culture system is an automated unit where buffered rumen fluid is incubated for up to 48 h with a substrate and a treatment, as described previously (Muetzel et al., 2011). Briefly, the system is running with an inoculum containing 20% v/v filtered rumen fluid in a carbonate based buffer (Mould et al., 2005). The substrate concentration is 10 mg/ml of inoculum incubated at 39°C where gas production is measured by the pressure in the headspace and methane and hydrogen emission are determined in a gas chromatography. Supplementary Figure 2 depicts the experimental design including biological and technical replicates. Samples were taken anaerobically and processed for metagenomic DNA sequencing. Metagenomic data was deposited under BioProject accession number PRJNA679310.

### Continuous Flow Rumen Fermenter

The continuous flow rumen fermenter was developed and set-up as described previously (PONE-D-21-05378, under review). In short, the system comprises a 1 L liquid volume single flow gravity outflow continuous culture system. The vessels are arranged in a linear array in a heated box, kept at 39°C with a cooled water bath (4°C) below in which the overflow is collected in bottles. Gas production is measured via a pressure sensor that also controls the liquid level of the vessel in combination with a liquid and a gas solenoid valve and methane and hydrogen emissions are determined via a dedicated gas loop through a gas chromatograph. The system also allows for the formation of a feed mat as the agitations of the fermentation contents are controlled by on/off cycles at various speeds and the liquid and solid inputs are also fully automated and programmable. The buffer and other liquids are delivered by dedicated computer-controlled pumps. The feed is also delivered computer controlled via a stepper motor mounted to a tube (Supplementary Figure 6).

Briefly, four individual fermenters were set up as follows. The liquid flow was 1.5 L/d and 24 g of feed were delivered in equal portions at 08.00 and 16.00 h. Gas sampling was set to a rate of 4 measurements per vessel and hour. Two vessels were supplemented with non-functionalized nanoparticles (control), and two vessels with dual-fusion PeiR nanoparticles (treatment). In addition, two vessels without nanoparticle addition were added to provide a baseline for gas measurements and potential nanoparticle degradation. Based on data obtained from the batch fermenter experiments, a dosage rate of 10 mg/ml of nanoparticles was chosen for the continuous flow rumen simulation. This dosage rate represented the optimal compromise between the ability to manufacture sufficient quantities of non-functionalized and biologically active PeiR dual-fusion nanoparticles and the anticipated loss of efficacy. The continuous flow fermenter was calibrated for 5 days to stabilize the rumen communities and supplemented with powdered ryegrass. Bioparticles were pulse-fed over 12 days twice daily to investigate the long-term impact of dual-fusion PeiR nanoparticles on the methanogen community. Samples for community analyses were taken from 1 day prior to treatment to day 12, while gas production was measured from day 1 of treatment to day 11. Metagenomic data was deposited under BioProject accession number PRJNA680790.

Immediately flushing the gas lines, the gas sample loop was switched in line with a gas chromatograph (GC) equipped with a MTX Plot column, a thermal conductivity and a flame ionization detector for the measurement of hydrogen and methane, respectively. The GC was run isothermic at 85°C. Calibration of the gas chromatography system was carried out by using gas alpha standards containing 2, 5, 10, and 20% methane and 1, 2.5, 5, and 10% hydrogen in nitrogen.

Short chain fatty acids were determined using a Hewlett-Packard (HP) 6890 series GC with an auto-injector and flame ionization detector. A Zebron ZB-FFAP 30.0 m × 0.53 mm I.D. × 1 μm film column was used for separation with a column helium flow rate of 5.5 ml/min. 1.0 μl of sample was injected into the column inlet (cool on-column inlet). The inlet temperature was set to track oven temperature and the detector was at 240°C. Column temperature was programmed to start at 85°C and increase by 10°C/min up to 180°C (held for 5 min), giving a run time of 14.5 min per sample. Peaks were quantified from standard curves of known concentrations of acetate, propionate, butyrate, and minor fatty acids (valerate, caproate, isobutyrate, and isovalerate).

### Archaeal Community Analyses

Frozen samples from the rumen batch or continuous flow fermenter were defrosted and a 200 μl sample taken with a wide bore pipette to ensure sampling of both the liquid and solid phases. Total DNA from 50 samples was extracted using a bead beating protocol, and archaeal and bacterial 16S rRNA genes were amplified as described previously (Kittelmann et al., 2013). For *in vitro* batch culture experiments, amplicons were quantified, pooled at equal concentrations, gel-purified and subjected to 454 Titanium amplicon sequencing at Macrogen, South Korea. For continuous flow rumen fermenter experiments, archaeal 16S rRNA gene regions were PCR amplified using and pooled for sequencing using an Illumina MiSeq 2x250base PE V2 at the Massey Genome Service (Palmerston North, New Zealand).

Sequence data was quality-filtered using default parameters in QIIME (Caporaso et al., 2010), and individual reads were mapped back to the corresponding samples by using the 12 bp error-correcting Golay barcodes. Archaeal 16S rRNA gene sequence reads were clustered into OTUs at 99 and 97% similarity using uclust (Edgar, 2010), respectively. Taxonomic assignment of archaeal and bacterial OTUs was done by BLAST using the RIM- (Seedorf et al., 2014) and Greengenes- (McDonald et al., 2012) database, respectively. Relative abundance tables were generated at the species level, and only species that contributed ≥ 1% to the total community in at least one sample were retained for the analysis. Principal coordinate analysis was carried out in QIIME using the Bray-Curtis dissimilarity metric.

### Quantitative PCR of Archaeal 16S rRNA Genes

Quantitative PCR (qPCR) of archaeal 16S rRNA was carried out as described earlier (Jeyanathan et al., 2011). Briefly, amplification of 16S rRNA gene fragments was carried out using the primer pair 109f (5′-ACKGCTCAGTAACACGT-3′) and 915r (5′-GTGCTCCCCCGCCAATTCCT-3′) using amplification parameters as described. qPCR was carried out in duplicate directly on undiluted and 1:10 dilutions of DNA, respectively, on a Rotorgene Realtime PCR machine (Qiagen, Hilden, Germany), using SYBR Green as the fluorescent indicator. Data was analyzed with the Rotorgene Q software, version 2.3.5.

### Statistical Methods

One Way ANOVA tests were carried out in SigmaPlot version 14.0 (Systat Software Inc.) with *P* < 0.050. Statistical significance for dose response and animal variance was determined using Restricted Maximum Likelihood (REML) variance components analysis with the following two optimization parameters: residual term and sparse algorithm with AI optimization added to the model. Approximate least significant differences of REML was set at 5%. In addition, a conservative multiple comparison Bonferroni test (*P* < 0.01) was carried out.

Multivariate data analyses were carried out to interrogate levels of animal-to-animal variation as well as dose effects across animals over time. The similarities of bacterial communities were explored using non-metric multi-dimensional scaling (NMDS) ordination in two dimensions (goodness of fit < 0.2). The Bray-Curtis similarity matrix was used to perform NMDS ordination using the relative abundance data. The relative association of phyla or genera to each of the treatment combination groupings was also explored using correspondence analysis (CA) based on the accumulated abundances. A matrix of similarity between treatment groupings and bacteria groups (phylum or genus) was constructed by projecting the bacteria group points onto the direction vectors of each treatment grouping point in the multidimensional CA configuration. These similarities or measures of association were then used to create clustered image maps (CIM) to highlight the relationship between the treatment groupings and the phyla or genera communities. All statistical analyses were carried out using the R software version 3.1.0. Data were visualized using OriginLab OriginPro 2021 version 9.8.0.200, Systat SigmaPlot version 14.0 and Microsoft Excel version 2008.

## Results

### Effectiveness of Lytic Enzyme Displaying Nanoparticles

**FIGURE 1:**
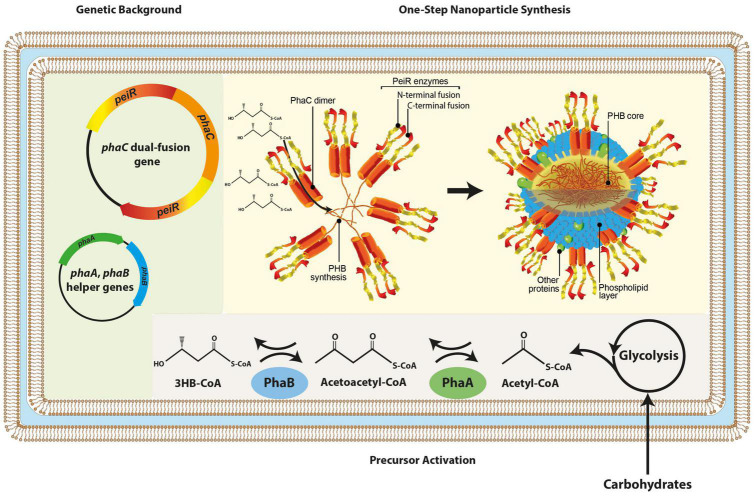
Schematic presentation of *in vivo* nanoparticle synthesis. The graphic representation depicts the genetic backbone (light green background) used for dual-fusion functionalized nanoparticles, subsequent redirection and activation of metabolic precursors (gray background) and the one-step nanoparticle biosynthesis where PeiR-PhaC-PeiR fusion dimers mediate PHB synthesis, a hydrophobic macromolecule auto-aggregating into nanoparticles. PeiR-PhaC-PeiR fusion dimers are directionally located on the bead surface (yellow background). Biosynthesis is carried out in *E. coli*. 3HB-CoA, 3-hydroxybutyryl coenzyme A.

A dual-fusion gene was synthesized, whereby *phaC* was flanked by two *peiR* genes, respectively, separated by a linker to enable greater steric flexibility. *In vivo*, PhaC acts as a dimer synthesizing PHB, providing the basis to display four PeiR enzymes in the same location on the nanoparticle surface. By presenting four PeiR enzymes in pairwise opposing directions, we predicted an increase in effectiveness of cell-wall binding to pseudomurein-presenting methanogens and subsequent lysis over the previously reported single fusion PeiR-PhaC nanoparticles (Figure 1).

*Methanobrevibacter* is the dominant taxonomic archaeal genus in the rumen (Vaidya et al., 2020) and *M. ruminantium* M1 and *M. gottschalkii* D5 have been selected as representative members of the rumen (Kittelmann et al., 2013). Comparing the efficacy of single- and dual-fusion PeiR displaying nanoparticles against rumen methanogens was achieved by analyzing short-term kinetics and sustained inhibition over time. Cell growth over time for both M1 and D5 was measured and a reduction (*P* < 0.05) in optical density against cultures supplemented with non-functionalized nanoparticles was observed after 30 min in M1 and 2 h in D5 (Supplementary Figures 1A,B), with dual-fusion nanoparticles consistently showing greater efficacy over the single fusion variety. While short-term kinetics quantified the ability to lyse a large number of cells quickly, it is equally important to determine the required concentration of PeiR-displaying nanoparticles for mediating prolonged inhibition of cell growth (minimal inhibitory concentration). Concentrations ranging from 0.02 to 1.00 mg/ml of dual-fusion nanoparticles were tested for their ability to inhibit cell growth (Supplementary Figures 1C,D) and methane production (Supplementary Figures 1E,F) over 3 days against M1 and D5, respectively. A minimal inhibitory concentration of 0.05–0.1 mg/ml was found to provide significant inhibition based on optical density against M1, while the minimal inhibitory concentration for D5 increased to 0.2 mg/ml (Supplementary Figure 1D). No methane production was observed for 1.0 mg/ml in both cultures after 3 days and this nanoparticle concentration was then used as a baseline for subsequent *in vitro* rumen simulations featuring a complex microbiota.

### Efficacy of Dual-Fusion PeiR Nanoparticles in a Rumen Batch Fermenter System

As a first step for testing the biological activity of dual-fusion PeiR nanoparticles in a more complex environment, rumen *in vitro* batch fermenters (Muetzel et al., 2014) were set up using freshly-sampled rumen fluid (Supplementary Figure 2).

#### Impact of Animal-to-Animal Variation on Archaeal Community Composition

Animal-to-animal and day-to-day variation in the rumen fluid used for inoculation of *in vitro* batch cultures can be a significant confounding factor as the starting communities in the inoculum collected vary in activity and, consequently, in metabolic output. Analysis of the inoculum from six cows without nanoparticle supplementation was carried out to investigate the degree of animal-to-animal variation. Principal component analysis of the total rumen archaeal community revealed that differences between cows often lead to distinct and separate clusters (Supplementary Figure 3). To verify that the changes in archaeal communities meditated by dual-fusion PeiR-nanoparticles can be detected above the observed animal variations, rumen fluid was treated with non-functionalized or dual-fusion PeiR-nanoparticles at a concentration of 34 mg/ml, and samples were taken at 0, 1, and 8 h. Although the composition of archaeal communities varied between animals at each given timepoint, trends in changes for the two major methanogen clades were consistent across animals (Supplementary Figure 4): a rapid decline in the *M. gottschalkii* clade within 1 h (∼70%), followed by a similar decrease in *M. ruminantium* populations. Across all cows, *M. ruminantium* and *M. gottschalkii* clades were significantly decreased by the addition of dual-fusion PeiR nanoparticles at 1 and 8 h (*P* < 0.01).

#### Changes in the Rumen Archaeal Communities Over Time

**FIGURE 2:**
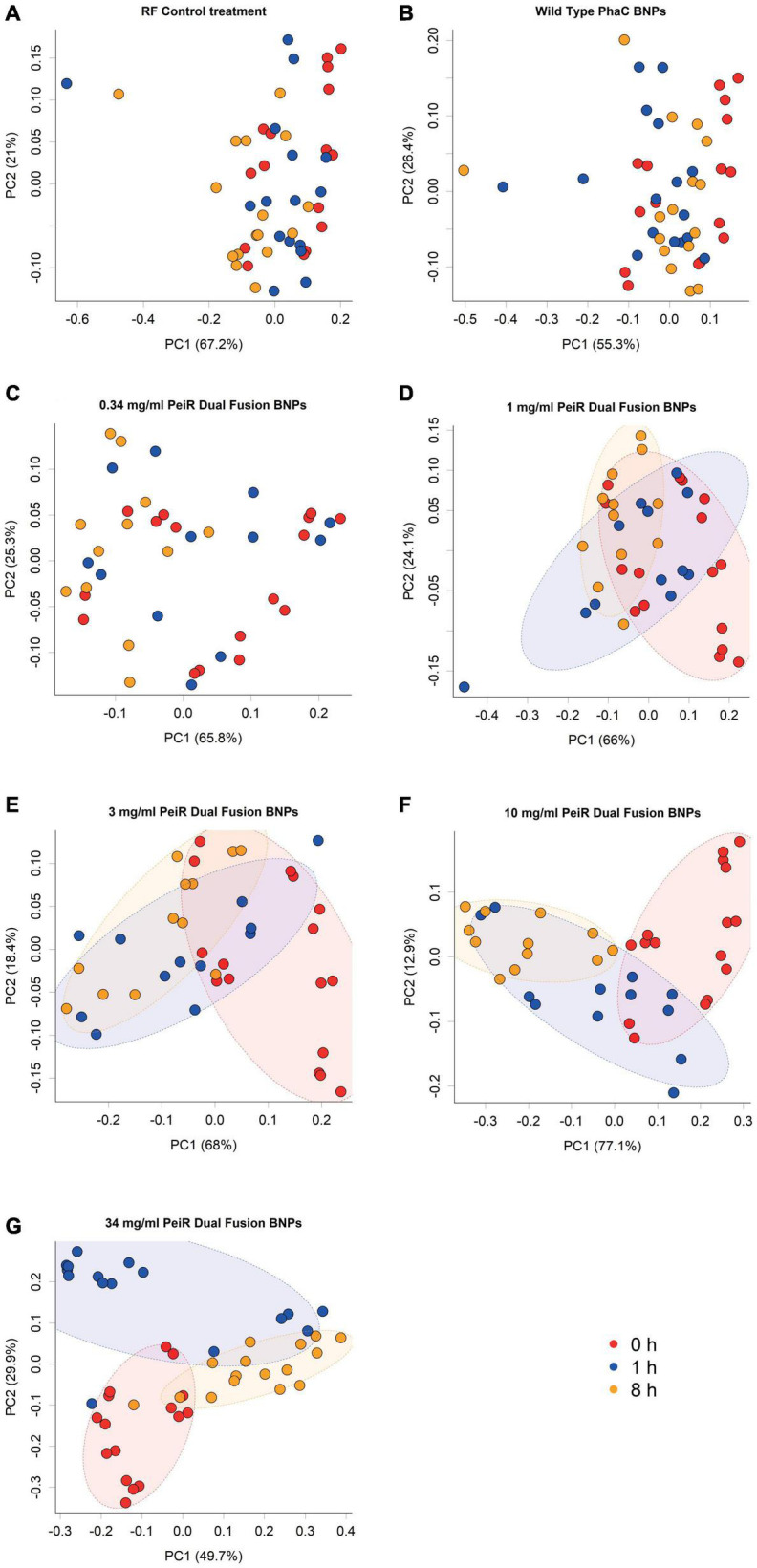
Principal component analysis of archaeal communities. Archaeal communities from *in vitro* batch cultivations were compared at different nanoparticle doses. The addition of non-functionalized nanoparticles did not lead to a change in community composition compared to the control **(A,B)**. Increasing concentrations of Dual-fusion PeiR nanoparticles **(C–G)** revealed a corresponding incremental shift in the community composition over 1 and 8 h. Colored dots represent individual measurements. Colors indicate sample time point (Red: t = 0 h; blue: t = 1 h; orange: t = 8 h). Colored ellipses indicate 75% confidence intervals.

A range of doses (0.34, 1, 3, 10, and 34 mg/ml) was chosen to account for an expected reduction in efficacy with the more complex microbiome in the rumen simulation. A principal component analysis of the rumen archaeal community composition revealed a dose-dependent longitudinal effect for dual-fusion PeiR nanoparticles (Figure 2). No separation was found for either control (rumen fluid only, RF) or non-functionalized nanoparticles at any time. With increasing concentrations of nanoparticles, growing shifts in the archaeal community compared to the control groups and in time were observed. At the highest dose of 34 mg/ml, a clear longitudinal separation emerged, separating baseline (0 h) from 1 and 8 h sampling points.

**FIGURE 3:**
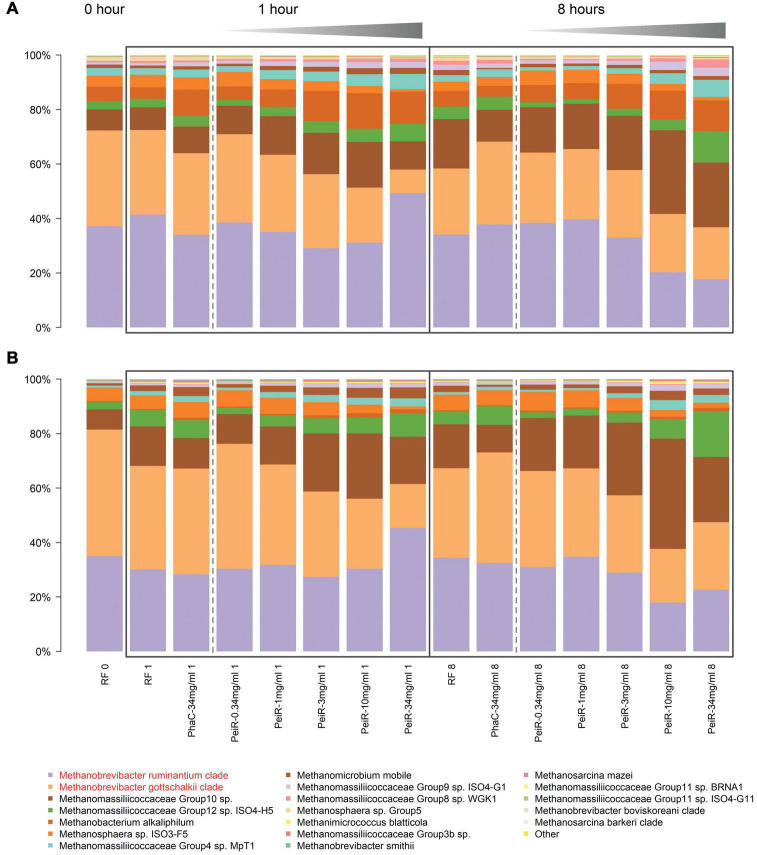
Relative changes in the archaeal community over time in a rumen batch model. *In vitro* batch fermentations were set up with rumen fluid inoculum sampled from two different cows, #7305 **(A)** and #39 **(B)**, respectively. The base state of the rumen archaeal community at t = 0 h (“RF 0”) is shown on the left. Control groups (Rumen Fluid only) and non-functionalized nanoparticles (“PhaC”) are presented at each sample time point. Non-functionalized nanoparticles were dosed at the highest corresponding dual-fusion nanoparticle concentration of 34 mg/ml. RF, Rumen fluid (control); PhaC, non-functionalized nanoparticles; PeiR, Dual-fusion PeiR nanoparticles. *M. ruminantium* (light purple) and *M. gottschalkii* (orange) clades are highlighted in red in the legend. Black boxes indicate the sample intervals of 1 and 8 h. Gray gradients visualize the increasing treatment dosages from 0.34 to 34 mg/ml. For each community profile, the top 20 most abundant taxa are presented, comprising > 99% of all taxa.

Detailed analyses of the relative archaeal community compositions supported observed trends of the principal component analysis (Figure 3). With increasing dual-fusion nanoparticle dose, relative abundances of the two major methanogen clades were decreased. Exposure to nanoparticles resulted in a dose-dependent initial reduction in the abundance of *M. gottschalkii* at 1 h which was then followed by a decrease in *M. ruminantium* at 8 h. While the relative abundance of *M. ruminantium* remained diminished, some recovery of *M. gottschalkii* could be seen at 24 h (data not shown), although this observation should be seen in light of possible nutrition depletion effects, buildup of gases and a reduction in pH control that occur in batch fermenter systems after extended fermentation times. Statistical significance of inhibition (*P* < 0.05) against *M. ruminantium* and *M. gottschalkii* compared to RF control was found for 3, 10, and 34 mg/ml doses at 1 h and for 10 and 34 mg/ml at hours 1, 8, and 24. Similarly, inhibition of *M. gottschalkii* was significant at concentrations of 3, 10, and 34 mg/ml at 1 h. Another observation related to the changes in the ratio of pseudomurein vs. non-pseudomurein-containing methanogens (Supplementary Figure 5). With increasing nanoparticle dosage rate, the relative abundance of pseudomurein-containing methanogens decreased, while those with a different cell envelope makeup such as Methanomassiliicoccales experienced an increase in their relative abundances. The growth of Methanomassiliicoccales is dependent on dietary methyl groups and may therefore be limited. These data support the hypothesis of functionalized nanoparticles as a fast-acting agent against methanogens within the boundaries of the rumen turnover for small particles (∼6–8 h).

### Degree and Duration of Changes in the Methanogen Population in a Continuous Flow Fermentation System

Although functionalized dual-fusion PeiR nanoparticles were demonstrated to be efficient inhibitors of rumen methanogens in batch fermentations, the dynamic environment of a rumen may pose a more challenging environment, featuring adverse effects such as washout of nanoparticles, non-specific adherence/adsorption by abiotic and biotic rumen components, degradation of nanoparticles and physical inaccessibility of rumen methanogens. To simulate the complex rumen environment, a continuous flow fermenter system was used to control pulse feeding of forage material, obtain real-time gas measurements, and sample the fermenting rumen content for subsequent microbiome analyses (Supplementary Figure 6).

#### Reactor Vessel Consistency

**FIGURE 4:**
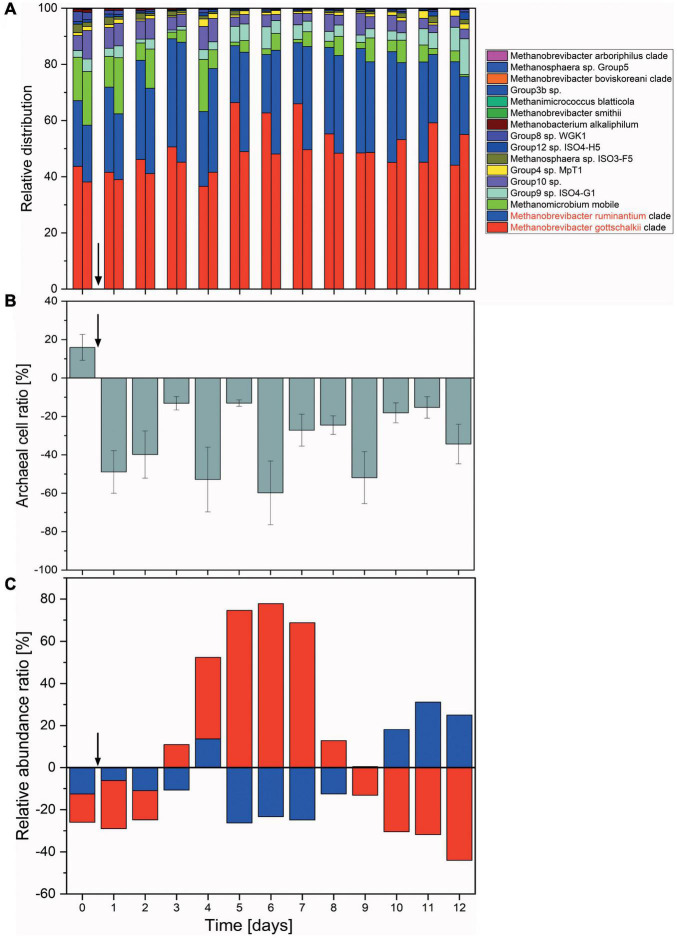
Changes in the archaeal communities in a continuous flow fermentation system. **(A)** Relative abundances shown as the sum of proportions of the archaeal community quantified by 16S rRNA gene DNA sequencing. For each day, respective left columns represent non-functionalized nanoparticles (control) and right columns dual-fusion nanoparticles. **(B)** Ratio of absolute archaeal numbers (copies/μl) compared between non-functionalized and dual-fusion PeiR nanoparticle treatments. Values represent the average of three measurements per day over two vessels. Error bars indicate standard errors. **(C)** Changes in the relative abundances of *M. ruminantium* (red) and *M. gottschalkii* (blue) as compared between non-functionalized and dual-fusion PeiR nanoparticles. Values are averaged over 24 h and two fermentation vessels. Vertical arrows indicate the start of nanoparticle treatments.

All fermentation vessels were inoculated with the same rumen fluid. However, these then turn into individual micro-environments with independent microbial succession patterns and subject to micro-changes in environmental conditions. While 5 out of 6 vessels reacted in a consistent and comparable way, one reactor vessel experienced a temporary disturbance where fermentation parameter values measured (e.g., butyric acid concentration) increased over the course of 3 days, only to return to a near-normal state at the end of the fermentation (Supplementary Figure 7). While this had a notable effect on short chain fatty acids concentrations, methane inhibition and archaeal numbers were much less affected (Figure 4), implying a disturbance within the bacterial community.

#### Methane Inhibition and Changes in the Archaeal Community Composition Over Time

**FIGURE 5:**
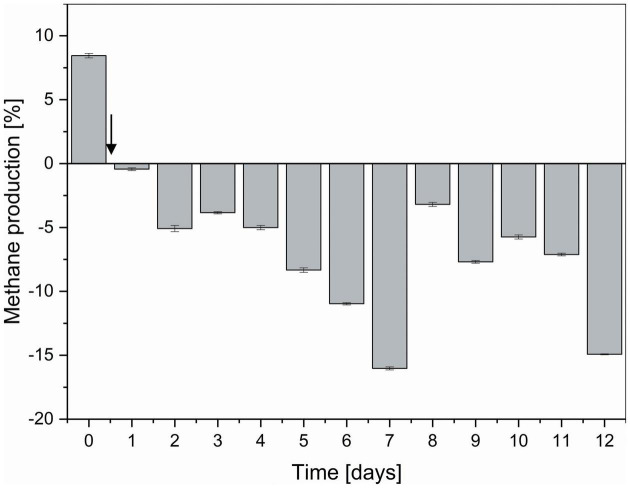
Degree of methane reduction in a continuous flow fermentation system. Methane inhibition as compared between non-functionalized and dual-fusion PeiR nanoparticles is expressed as percent inhibition over the course of 12 days. The vertical arrow indicates the start of nanoparticle addition to reactor vessels. Error bars represent the standard errors of two reactor vessels with 96 daily methane measurements each.

Compared to non-functionalized controls, addition of dual-fusion PeiR nanoparticles resulted in a 5–15% reduction in methane (as percent of total gas production, Figure 5). While a persistent methane inhibition was observed, the degree of inhibition oscillated over the course of the fermentation. This oscillation mirrors changes observed in the archaeal community profiles. None of the methanogen clades experienced a total collapse with the addition of dual-fusion PeiR nanoparticles for the duration of the fermentation (Figure 4A). While this was in line with the hypothesis of a single enzyme leading to subtle changes, on average a ∼30% reduction in total numbers of archaea compared to non-functionalized nanoparticles was detected (Figure 4B). Over the course of the fermentation, total numbers of archaea fluctuated by a factor of 5 (Supplementary Figure 8). Lower numbers of total archaea did not correlate with methane concentration, implying that changes to specific methanogen groups may be an important factor. Such an interplay can be observed between the two dominant rumen methanogen clades, *M. ruminantium* and *M. gottschalkii* (Figure 4C) where an oscillation occurs in the relative abundances between the two clades following addition of dual-fusion PeiR nanoparticles. Reduction in abundance in one clade coincides with a lower methane production (days 2–6 post-nanoparticle addition). As the first oscillation closes (day 7), methane production increases, only to drop again as the second oscillation takes place (days 8–12).

#### Changes in Short Chain Fatty Acid Concentrations

Key nutrients for the animal derived from rumen fermentation are short chain fatty acids (SCFAs), namely acetate, propionate and butyrate (Shen et al., 2017). The biological nanoparticles presented here are comprised of PHB, both synthesized and broken down again by a large number of microbes (Kliem et al., 2020). The presence of PHB nanoparticles in the rumen was hypothesized to lead to their degradation and release of energy to the ruminant in the form of butyrate. A first indication of such breakdown was observed in rumen batch fermenters which exhibited strong foaming upon nanoparticles supplementation (Supplementary Figure 9).

**FIGURE 6:**
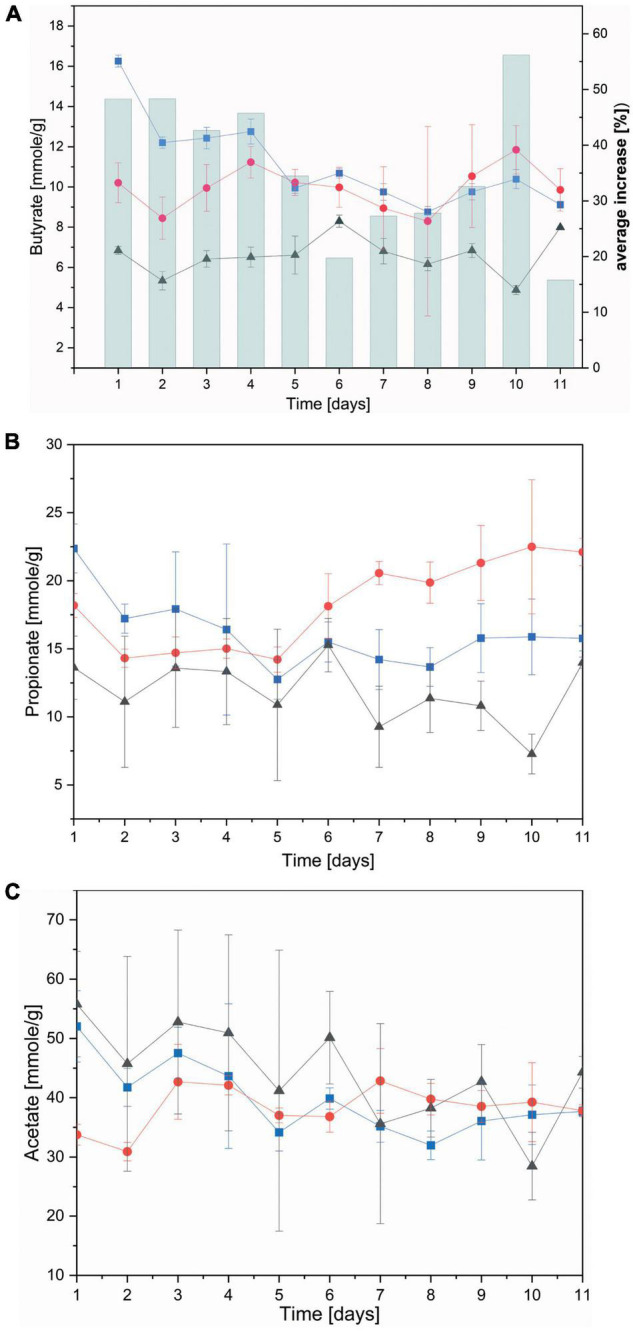
Concentration of short chain fatty acids. Short chain fatty acids were quantified as the average between two fermenter vessels in a continuous flow fermentation system. **(A)** Butyrate; bars indicate the (%) daily average increase of butyrate compared to nanoparticle-free control fermentation; **(B)** propionate; **(C)** acetate. Black: control without nanoparticles added, Blue: non-functionalized nanoparticles, Red: dual-fusion PeiR nanoparticles. Error bars indicate standard errors as averaged over days and fermentation vessels.

Subsequently, individual SCFAs were measured over the course of the continuous flow fermentation. Both non-functionalized and dual-fusion PeiR nanoparticles lead to a persistent increase in butyrate (average of 36% increase) and, to a lesser extent, propionate over RF control (Figures 6A,B). Addition of PHB nanoparticles, however, did not result in a change in acetate levels (Figure 6C). No decrease below pH 6.0 was observed during the continuous flow fermentation in any of the fermentation vessels (Supplementary Figure 10).

## Discussion

Here we present a new strategy, combining biotechnologically scalable PHB nanoparticle production with phage therapy concepts in a one-step *in vivo* biosynthesis.

The main rumen methanogen species comprise *Methanobrevibacter gottschalkii*, *Methanobrevibacter thaueri*, *Methanobrevibacter smithii*, and *Methanosphaera stadtmanae* (Patra et al., 2017; Moissl-Eichinger et al., 2018) and, in New Zealand ruminants Methanobacteriales and Methanomassiliicoccales comprise 99.98% of all rumen methanogens (Seedorf et al., 2015) and contribute up to 4% to the total rumen microbiota (Janssen and Kirs, 2008). Previously, we demonstrated that a methanogen virus lytic enzyme from *Methanobrevibacter ruminantium* M1, PeiR, is an effective agent inhibiting a range of rumen methanogen strains in pure culture, specifically against Methanobacteriales (Altermann et al., 2018). However, it was also recognized that this specific lytic enzyme features several drawbacks that may lead to a loss of function in more complex systems. PeiR is only biologically active in a reduced state, requiring more complex production, storage and treatment application. Furthermore, PeiR-functionalized nanoparticles self aggregate, leading to large clusters that reduce enzyme activity. To partially compensate the predicted loss in activity, a dual-fusion PeiR nanoparticle was designed, displaying two PeiR enzymes per PhaC synthase unit instead of one as previously described. PhaC acts as a dimer, which generates localized clusters of four PeiR enzymes, that are displayed pairwise in a directional manner. As hypothesized, dual-fusion PeiR nanoparticles outperformed their single-fusion counterparts and were taken forward toward rumen simulations.

A second consideration was the predicted variation in between batch and continuous flow fermenter vessels once these were inoculated and fermentation began. In particular the continuous flow-fermenter highlighted that a small pertubation in fermentaion pathways can lead to a different fermentaion outcome. The relatively small number of reactor vessels is a recognized limitation of the system that restricts statistical analyses and power, which must be weighed against its advantages of frequent sampling, greater control of environmental conditions and the avoidance of animal experiments.

The main hypothesis was that the addition of a single enzyme active against a specific group of methanogens is capable of quantitatively reducing methane emissions. Dual-fusion PeiR nanoparticles confirmed this hypothesis by mediating up to 15% reduction in emissions against non-functionalized nanoparticle controls over 11 days of continuous fermentation—but the effect on methanogens was more complex than anticipated. Instead of a simple reduction, an intricate interplay emerged between the two major rumen methanogen clades, *M. ruminantium* and *M. gottschalkii*. Over the course of the rumen simulation, inverse oscillations in relative abundances between these two clades were observed, seemingly associated with the longitudinal modulation in methane emissions. An oscillating pattern in methanogen populations in response to an inhibitor has not been reported previously. By extending these dynamic population patterns to other methanogen groups, a more differentiated approach to ruminant methane inhibition may be feasible without the abrupt changes to the rumen microbiome seen with other inhibitors (Martinez-Fernandez et al., 2016). Targeting other methanogens could be mediated through the deployment of nanoparticles displaying different bacteriophage lytic enzymes, all with their own host specificity and activity, opening the door for a plethora of functionalized nanoparticles, tailored toward different microbes, feeding regimes, and environmental drivers.

One of the key constraints for every ruminant methane mitigation strategy is to preserve—or enhance—animal productivity as part of the treatment. Short chain fatty acids, one of the main products of rumen microbial fermentation, deliver 50–70% of the total energy requirement of the animal (Shen et al., 2017). The increase of butyrate throughout the rumen simulations as a consequence of PHB nanoparticle addition, suggests a nanoparticle breakdown and provision of additional energy to the animal. The observed increase in propionate for dual-fusion PeiR nanoparticles was not correlated to changes in the archaeal community and may therefore reflect a modulation in bacterial composition or metabolism. Further, the implied breakdown of PHB nanoparticles in the rumen reduces or eliminates the accumulation of such agents in the environment post-digestion, creating an environmentally clean methane mitigation option.

While presently functionalized nanoparticles would be delivered in the form of a feed supplement, future opportunities may include the development of transgenic forage plants synthesizing a range of different functionalized nanoparticles, eliminating the need for biotechnological manufacture and delivery to the animal. These forage plants could be planted based on desired functionality (e.g., methane mitigation, pathogen inhibition), or forage quality (e.g., enhanced plant fiber digestion in drought conditions).

Here, we deliver the proof-of-principle of a new biotechnology-based methane mitigation strategy and applications will likely include other, more accessible lytic enzymes enabling the fine-tuned modulation not only of rumen methanogens but also of the rumen bacterial population, optimizing rumen fermentation and animal productivity for different feeding regimes and environmental pressures. Phage endolysins are modular in makeup and chimeric lysins can be rapidly created with changed host specificity and activity (Yang et al., 2017), reducing development cycles for new anti-methanogen agents.

## Data Availability Statement

The datasets presented in this study can be found in online repositories. The names of the repository/repositories and accession number(s) can be found below: https://www.ncbi.nlm.nih.gov/genbank/, PRJNA679310; https://www.ncbi.nlm.nih.gov/genbank/, PRJNA680790.

## Author Contributions

EA conceived and designed the project, with input from RR and SM. KR and SM performed the experiments and analyzed the data. WY analyzed and visualized metagenomic community data with input from EA. RR helped designing and carrying out pure culture experiments. EA and all other authors discussed the data and wrote the manuscript. All authors contributed to the article and approved the submitted version.

## Conflict of Interest

EA, RR, SM, KR, and WY were employed by AgResearch Ltd.

## Publisher’s Note

All claims expressed in this article are solely those of the authors and do not necessarily represent those of their affiliated organizations, or those of the publisher, the editors and the reviewers. Any product that may be evaluated in this article, or claim that may be made by its manufacturer, is not guaranteed or endorsed by the publisher.

## Footnotes

1
https://www.thermofisher.com/nz/en/home/life-science/cloning/gene-synthesis.html
