# Supplementary material for: Tailored Nanoparticles With the Potential to Reduce Ruminant Methane Emissions
Source: Front Microbiol. 2022 Mar 11;13:816695. doi: 10.3389/fmicb.2022.816695 (PMC8963448; doi:10.3389/fmicb.2022.816695)
Supplement: Supplementary file 1 [file Data_Sheet_1.docx]

Supplementary Material

# Supplementary figures


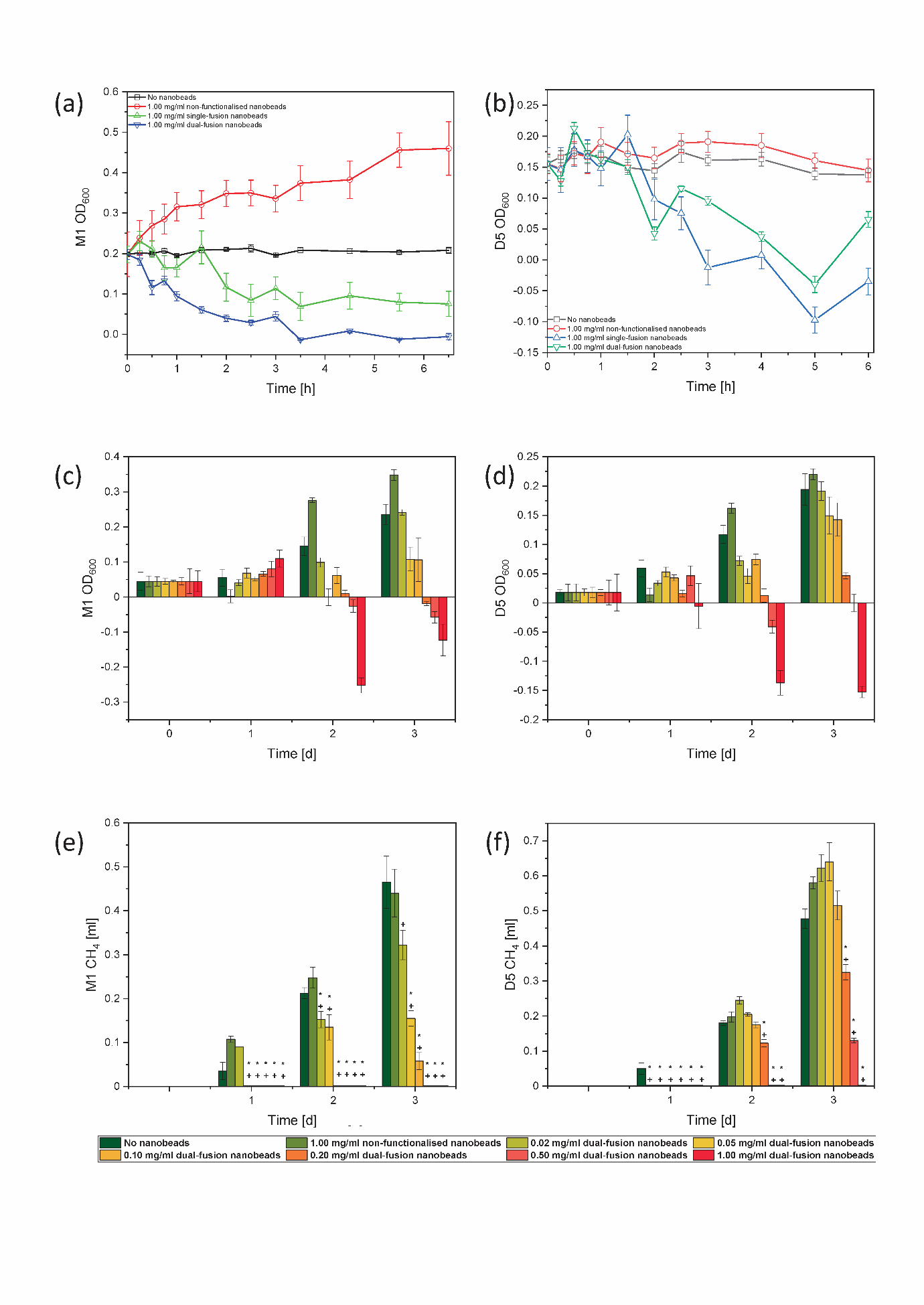


**Supplemental Figure 1:** Short term kinetics of single (PeiR-PhaC) and dual (PeiR-PhaC-PeiR) fusion nanoparticles against the host strain *M. ruminantium* M1 (a) and *M. gottschalkii* D5 (b). OD_600_ was measured corrected for as previously described*^32^*. ‘Dual-fusion nanoparticles’ were statistically different (P < 0.05) against the PhaC non-functionalised nanoparticle control for M1 from t >= 0.5 h and from t >= 2 h for D5, albeit at a higher nanoparticle concentration. Minimal inhibitory concentrations were measured via optical density for M1 (c) and D5 (d) with dual-fusion nanoparticle concentrations ranging from 0.02 mg/ml to 1.00 mg/ml. Similarly, levels of methane in the culture headspace was measured for M1 (e) and D5 (f). Error bars represent standard errors. An asterisk indicates a statistically significant difference (P < 0.05) against the PhaC control nanoparticles and a cross against the respective pure culture without nanoparticle addition. The slow cell growth of methanogens introduced a greater level of variability for the first 24 hours.


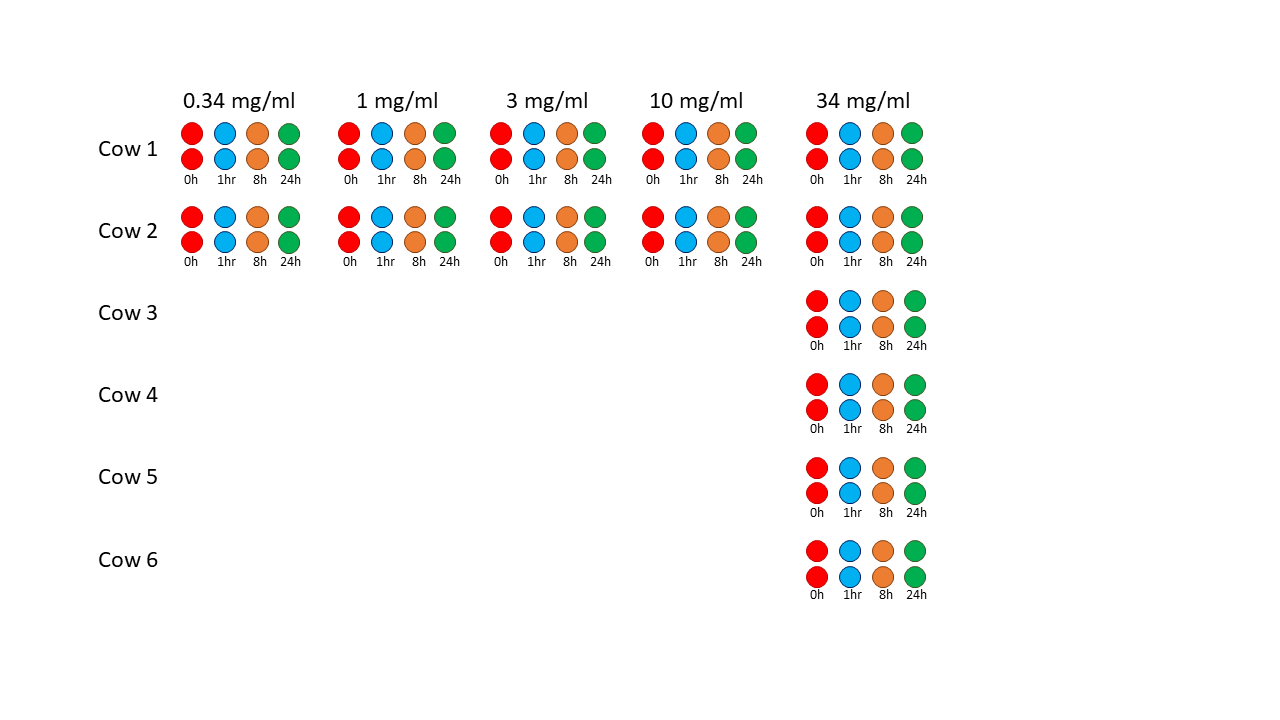


**Supplemental Figure 2**: Optimised experimental design to evaluate animal-to-animal variation and effect of dosage on the methanogen community when subjected to WT and dual-fusion PeiR-BNPs. Each dot represents an individual sampling event. 6 biological replicates (cows 1 - 6) with two technical replicates (two corresponding dots of the same colour) for each time point sampled (animal-to-animal variation). Two biological replicates (cows 1 and 2) for each BNP dosage rate tested with two technical replicates for each time point sampled (dose effect).


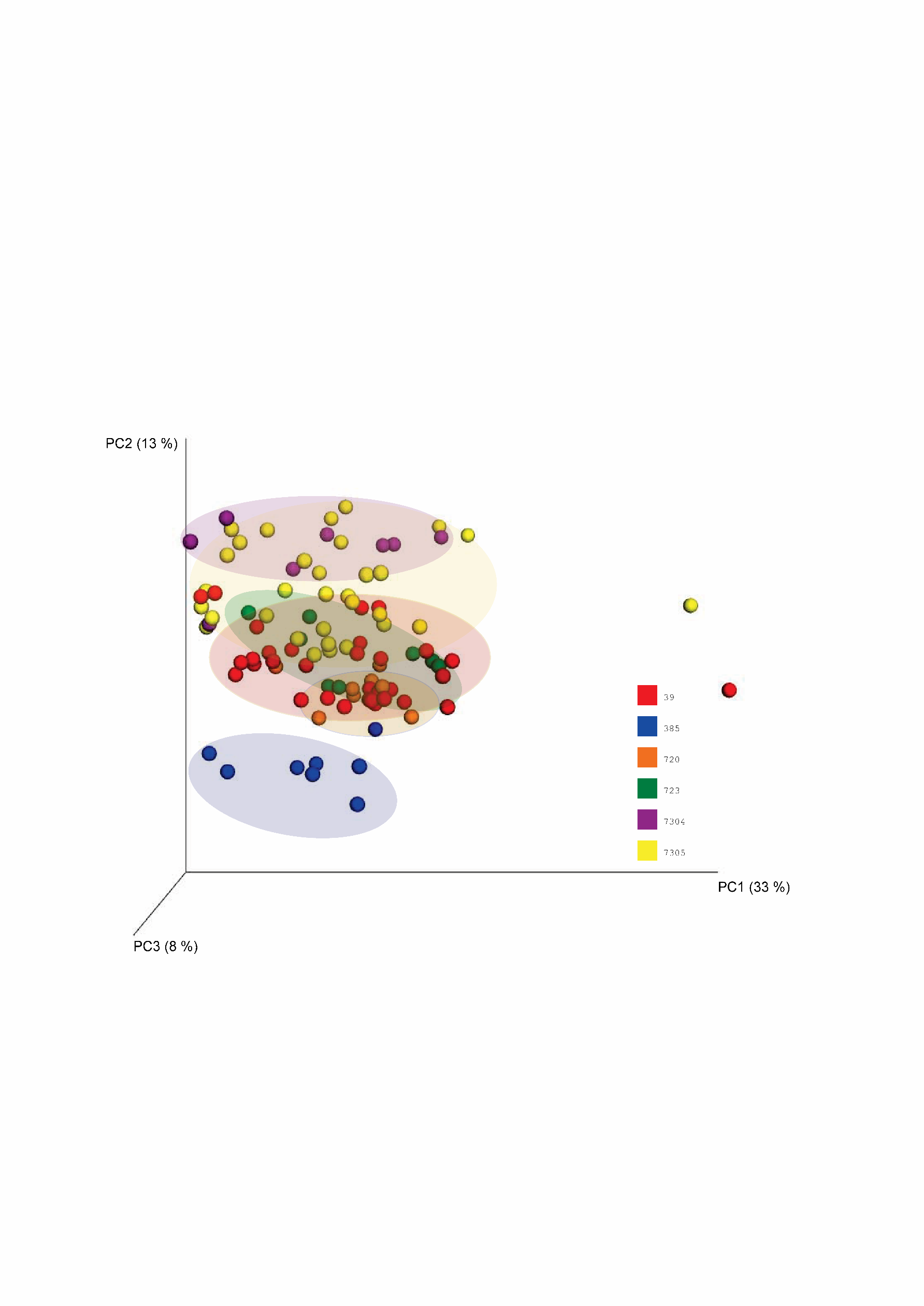


**Supplemental Figure 3**: Principal component analysis of the rumen archaeal community without nanoparticle addition at times 0, 1 and 8 hours provides a baseline view of the differences between the six fistulated cows used in this experiment. Individual cows are represented by different colours as indicated in the legend. Each dot represents a rumen sample.

*
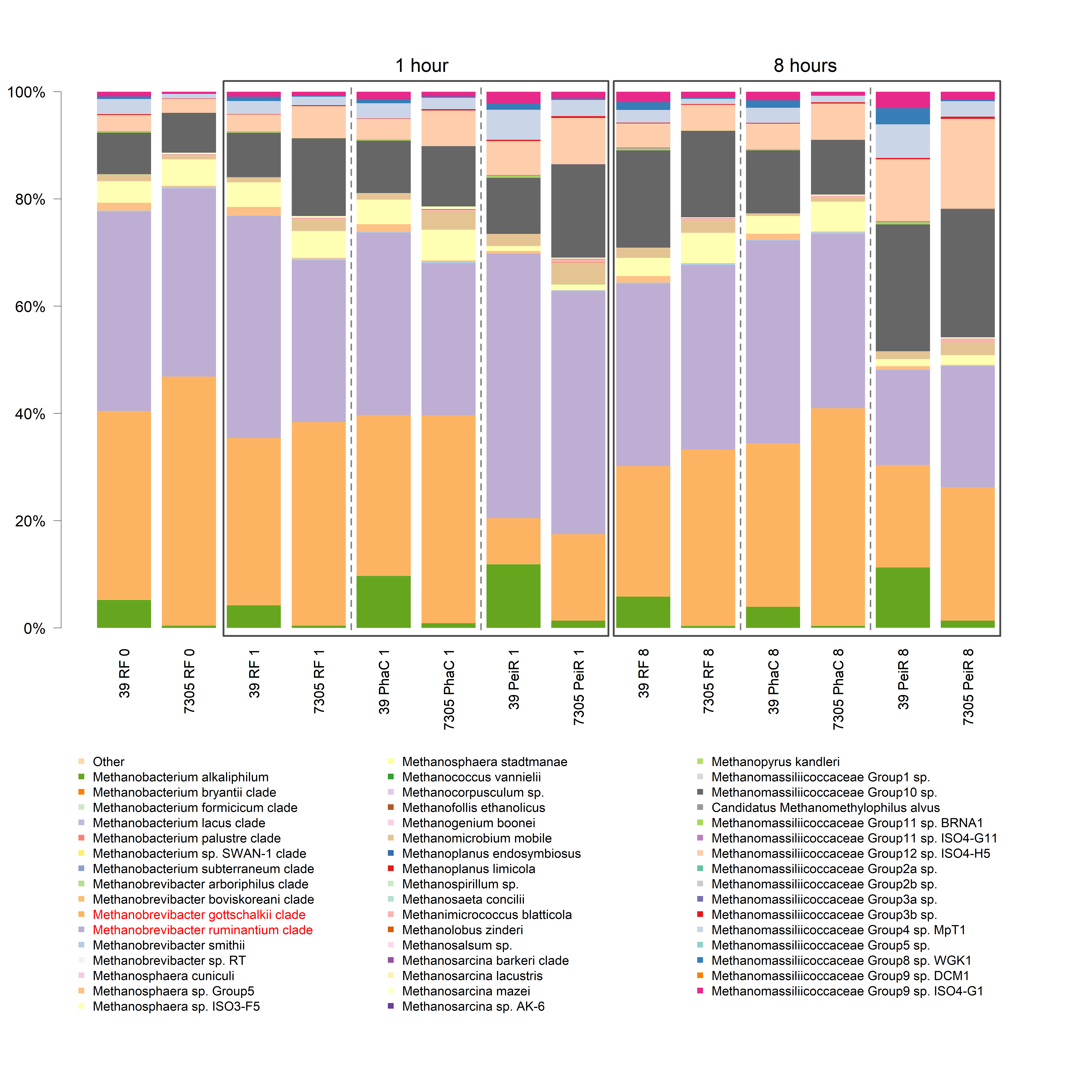
*

**Supplemental Figure 4***:* Representative relative distribution of archaeal taxa for cows 39 and 7305. Data for the remaining 4 cows revealed the same trends but were omitted here to reduce the complexity of the figure. RF = Rumen Fluid (control); PhaC = non-functionalised nanoparticles; PeiR: dual-fusion PeiR nanoparticles. *M. ruminantium* (gold) and *M. gottschalkii* (grey) clades are highlighted in red in the legend. Black boxes indicate the sample intervals of 1 and 8 hours. The baseline state of the rumen archaeal community for both cows at t = 0 h is shown on the left. RF control, non-functionalised control and dual-fusion PeiR nanoparticles treatments are shown individually at each sample time point for both cows.


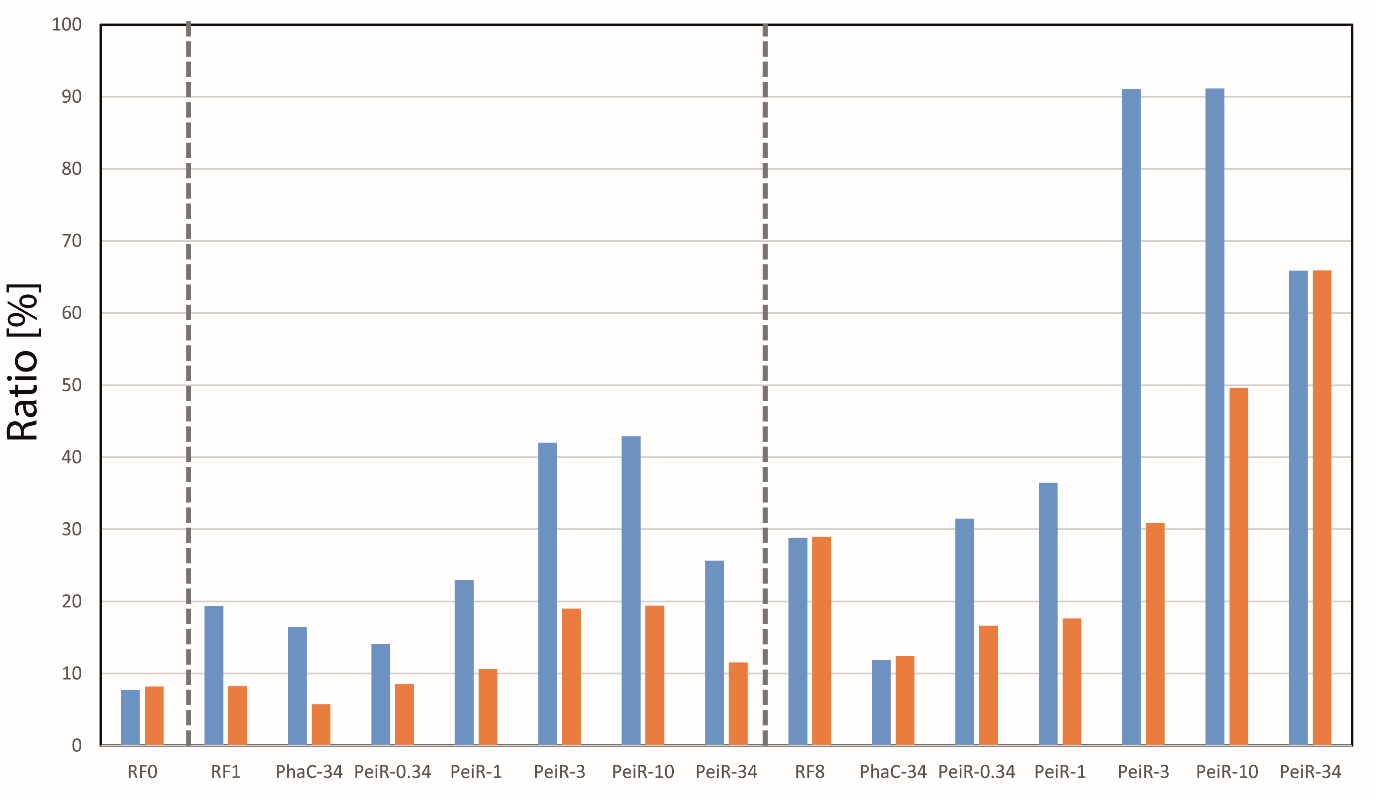


**Supplemental Figure 5**: Ratios of relative abundances between pseudomurein and non-pseudomurein containing methanogens in a rumen batch model. Blue and red bars represent ratios for cows 7305 and 39, respectively. RF0, RF1 and RF8 indicate rumen fluid only controls at times 0, 1 and 8 hours. PhaC depicts non-functionalised nanoparticle controls provided at the highest dosage rate of 34 mg/ml. PeiR indicates dual-fusion nanoparticle treatment at dosage rates of 0.34, 1, 3, 10 and 34 mg/ml. Vertical dashed lines separate sampling time groups.

**
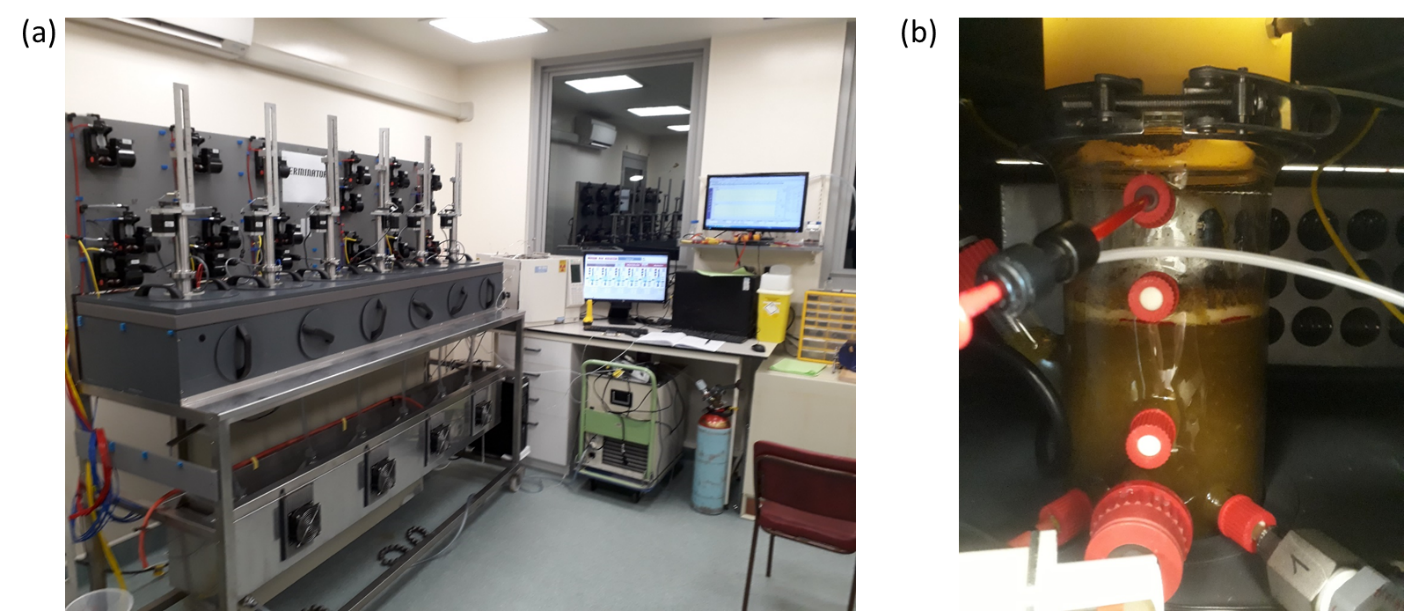
**

**Supplemental Figure 6**: Continuous flow rumen fermenter. (a) Up to six individual reactor vessels can be inoculated with freshly sampled rumen fluid in parallel, supplemented with artificial saliva. Monitoring of gases in the reactor vessel headspace is automated via automated sampling directly fed into a gas chromatograph. (b) Individual reactor vessel showing and ongoing rumen fermentation, feed and inlet ports on the bottom of the vessel and a gas port on the top.


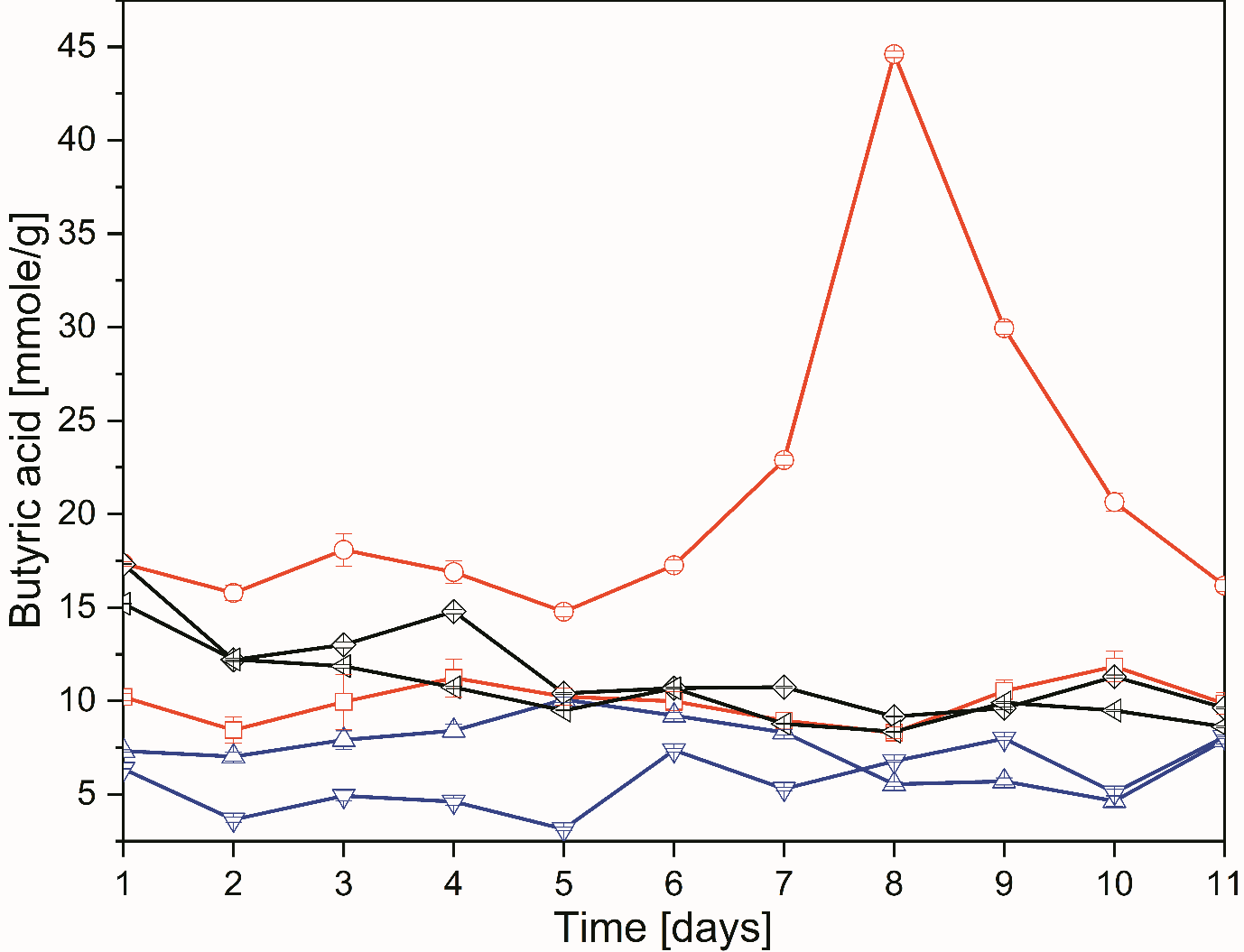


**Supplemental Figure 7**: Example of reactor vessel consistency and disturbance during an 11 day continuous fermentation. The graph depicts the concentration of butyric acid present in the rumen fluid. Each data point represents the average of three daily measurements. Error bars represent standard errors. Black: internal control, no nanoparticles added. Blue: non-functionalised nanoparticles. Red: dual-fusion PeiR nanoparticles.


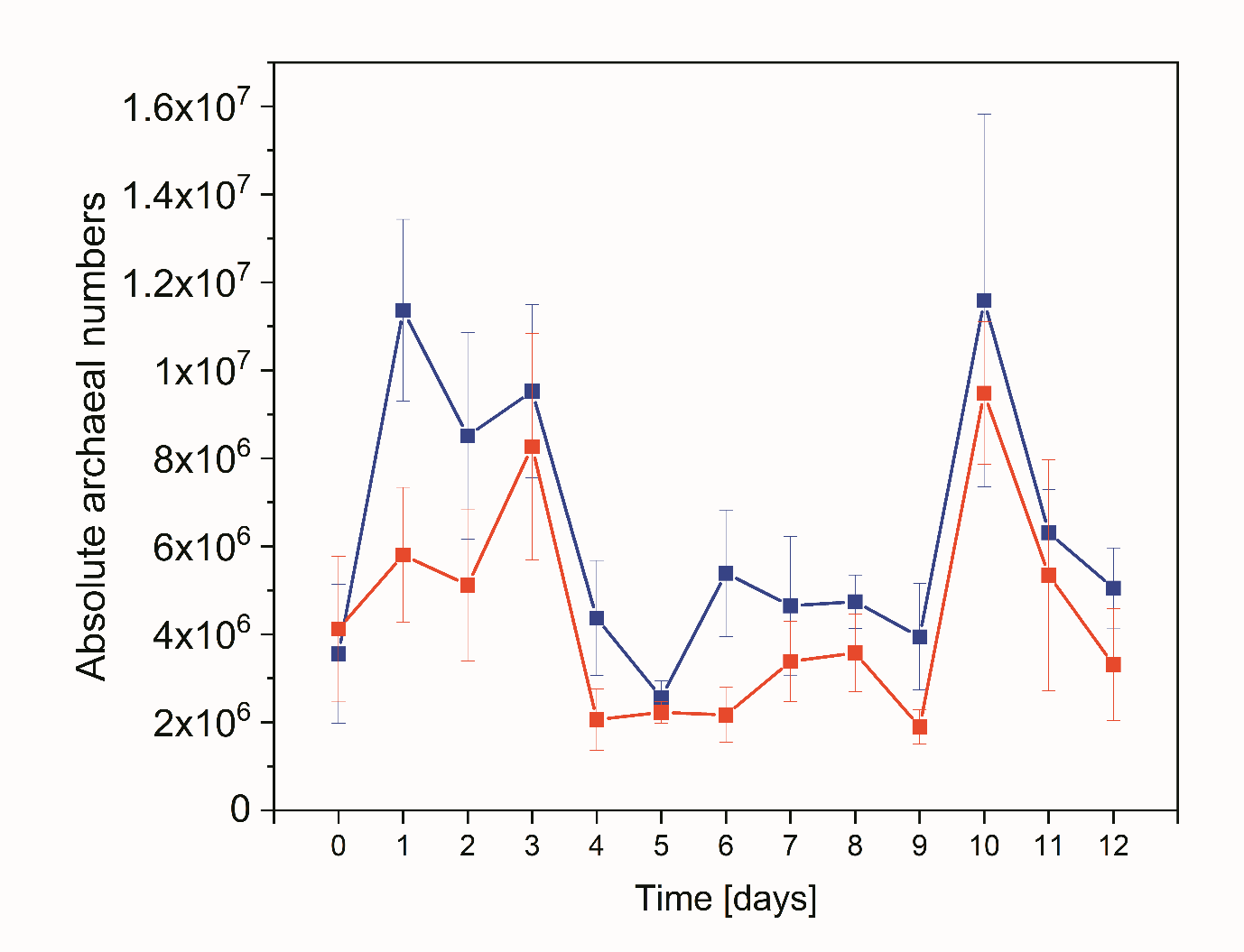


**Supplemental Figure 8**: Absolute archaeal numbers for non-functionalised (blue) and dual-fusion PeiR (red) nanoparticle treatments. Values represent the average of three measurements per day over two vessels. Error bars indicate standard errors.


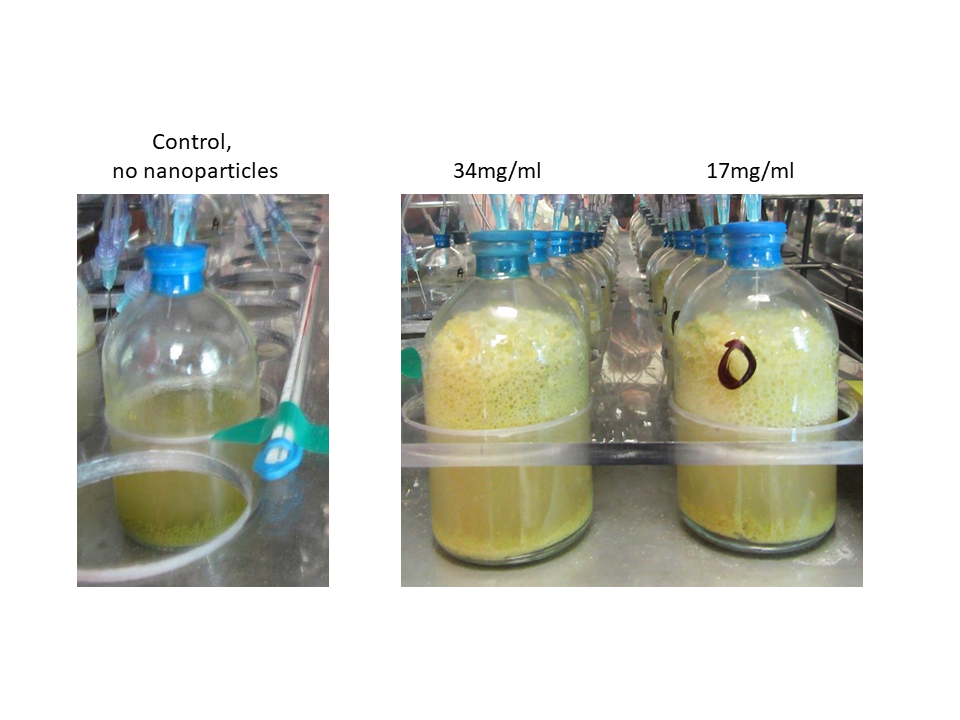


**Supplemental Figure 9**: Rumen batch fermenters supplemented with PHB nanoparticles at concentrations of 0, 17 and 34 mg/ml.


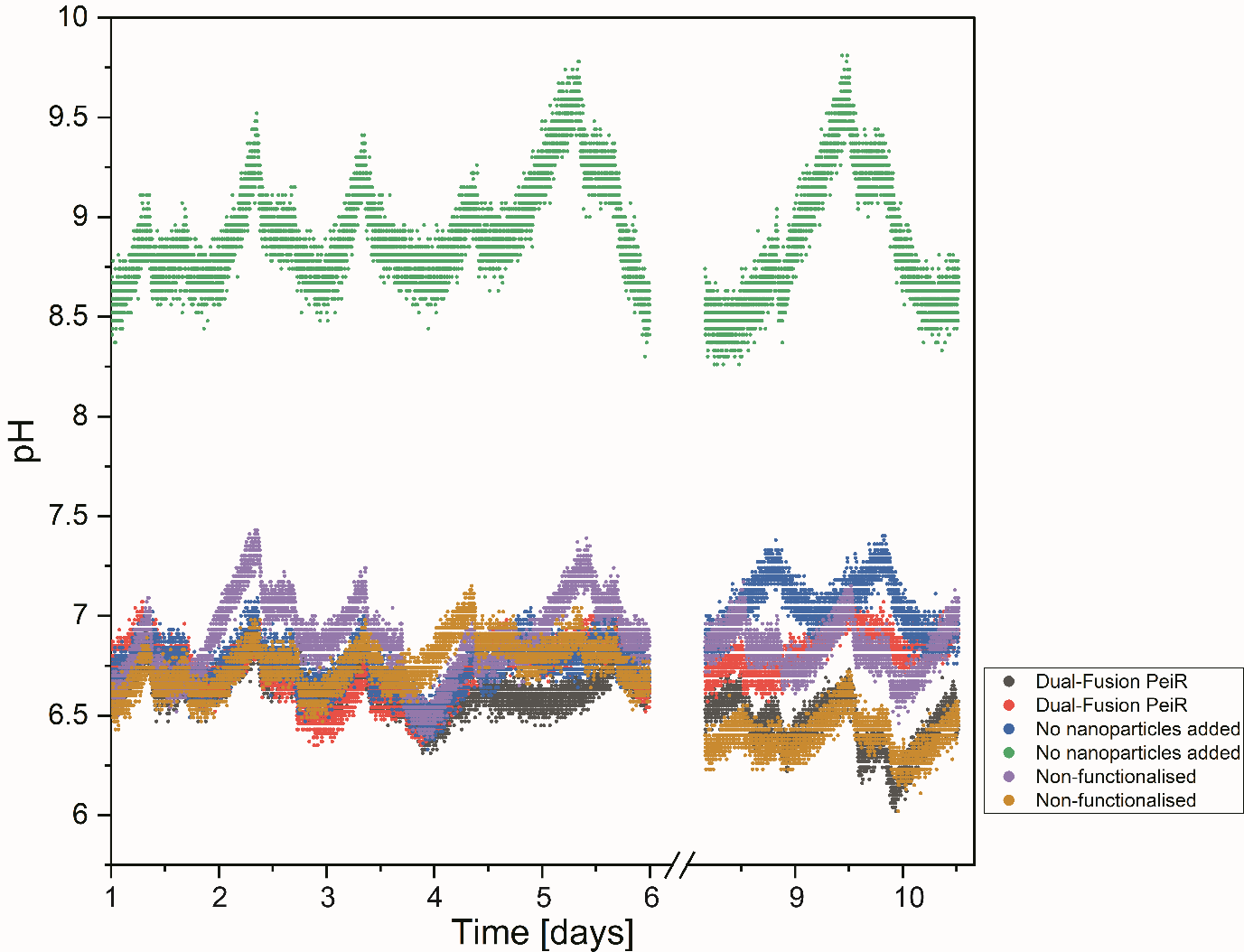


**Supplemental Figure 10**: pH over time across continuous flow reactor vessels. A probe fault occurred during the run on days 7 and 8 and resulted in a loss of data. However, during the measured timepoints, none of the vessels dropped below pH 6.0 which reduces the likelihood of onset of rumen acidosis, a metabolic disease state where the rumen pH drops below pH 5.5 that, in severe cases, can lead to increased animal mortality.
